# Src Family Kinases and p38 Mitogen-Activated Protein Kinases Regulate Pluripotent Cell Differentiation in Culture

**DOI:** 10.1371/journal.pone.0163244

**Published:** 2016-10-10

**Authors:** Boon Siang Nicholas Tan, Joly Kwek, Chong Kum Edwin Wong, Nicholas J. Saner, Charlotte Yap, Fernando Felquer, Michael B. Morris, David K. Gardner, Peter D. Rathjen, Joy Rathjen

**Affiliations:** 1 School of BioSciences, University of Melbourne, Parkville, Australia; 2 Stem Cells Australia, The University of Melbourne, Parkville, Australia; 3 Australian Stem Cell Centre, Monash University, Clayton, Australia; 4 Menzies Institute of Medical Research, University of Tasmania, Hobart, Australia; 5 School of Molecular and Biomedical Science, University of Adelaide, Adelaide, Australia; 6 School of Medicine, University of Tasmania, Hobart, Australia; Laboratoire de Biologie du Développement de Villefranche-sur-Mer, FRANCE

## Abstract

Multiple pluripotent cell populations, which together comprise the pluripotent cell lineage, have been identified. The mechanisms that control the progression between these populations are still poorly understood. The formation of early primitive ectoderm-like (EPL) cells from mouse embryonic stem (mES) cells provides a model to understand how one such transition is regulated. EPL cells form from mES cells in response to l-proline uptake through the transporter Slc38a2. Using inhibitors of cell signaling we have shown that Src family kinases, p38 MAPK, ERK1/2 and GSK3β are required for the transition between mES and EPL cells. ERK1/2, c-Src and GSK3β are likely to be enforcing a receptive, primed state in mES cells, while Src family kinases and p38 MAPK are involved in the establishment of EPL cells. Inhibition of these pathways prevented the acquisition of most, but not all, features of EPL cells, suggesting that other pathways are required. L-proline activation of differentiation is mediated through metabolism and changes to intracellular metabolite levels, specifically reactive oxygen species. The implication of multiple signaling pathways in the process suggests a model in which the context of Src family kinase activation determines the outcomes of pluripotent cell differentiation.

## Introduction

The pluripotent cell lineage in the mouse embryo is founded in the forming blastocyst and develops through a series of functionally distinct intermediate populations before differentiating at gastrulation. Four identifiable pluripotent cell populations, or states, have been identified *in vivo*–the epiblast precursor cell, the epiblast of the Inner Cell Mass (ICM), and the early and late epiblast of the post-implantation embryo [1–3]. Culture equivalents of these populations have been established from mouse. Primed embryonic stem (mES) cells [4, 5] and epiblast stem cells (EpiSCs) [6–8], which represent the epiblast of the ICM and the late epiblast of the post-implantation embryo, respectively, have been isolated directly from the embryo. Naive ES cells, an *in vitro* equivalent of the early epiblast of the ICM, have been formed from primed mES cells in culture [9–12]. Lastly, the epiblast, or primitive ectoderm, of the early post-implantation embryo can be formed in culture through the differentiation of primed mES cells to early primitive ectoderm-like (EPL) cells [13],[14, 15]. EpiSC-like cells can also be derived from mES cells by culture in FGF and Activin A [16–18]. These populations of pluripotent cells are now well recognized, but the molecular mechanisms that regulate progression between them are not well understood.

EPL cell formation occurs when mES cells are cultured in MEDII, medium conditioned by HepG2 cells [13–15], or in medium containing the active component of MEDII, l-proline [19–22]. Expression of *Oct4*, *Sox2* and alkaline phosphatase, and a differentiation potential in culture that includes mesoderm, endoderm and ectoderm, identifies EPL cells as pluripotent [13, 23–28]. The changes in colony morphology, gene expression [13, 14, 24, 29], proliferation rate [20], and developmental potential [15, 24, 25] that accompany EPL cell formation identify these cells as primitive ectoderm-like. EPL cell formation is dependent on elevated concentrations of l-proline within the medium (> 100 μM) [19, 20], and is inhibited by LIF [13]. The uptake of l-proline through the sodium-coupled neutral amino acid transporter 2 (Slc38a2, also known as SNAT2) on the surface of the cells is required for activity, and the inhibition of l-proline uptake through SNAT2 prevents EPL cell formation [19]. Collectively, these studies describe a system that models the transition from the epiblast of the ICM to early primitive ectoderm, and that can be used to understand the regulation of this event.

Little is known of how the internalization of l-proline by ES cells, when presented in MEDII or added exogenously, induces EPL cell formation. Changes in cell morphology characteristic of the system have been shown to require the metabolism of l-proline and generation of reactive oxygen species (ROS) [21]. Here we consider the role of signaling pathways in EPL cell formation. We describe the effect of pharmacologically inhibiting the Src family kinases and mitogen-activated protein kinase (MAPK) pathways (p38 MAPK and Extracellular signal-regulated kinases 1 and 2 (ERK1/2)) on the formation and maintenance of EPL cells. We show that inhibition of Src family kinases and p38 MAPK pathways, and pathways implicated in naïve cell formation, affected the formation and maintenance of EPL cells. Inhibition of a single pathway could not completely prevent EPL cell formation, suggesting the requirement of multiple signaling pathways in the process. These data have been used to develop a model for the process of pluripotent cell lineage progression and the formation of the primitive ectoderm based on a metabolic switch and increasing intracellular ROS.

## Results

### Inhibition of ERK1/2 signaling prevents EPL cell formation and maintenance in culture

**Fig 1:**
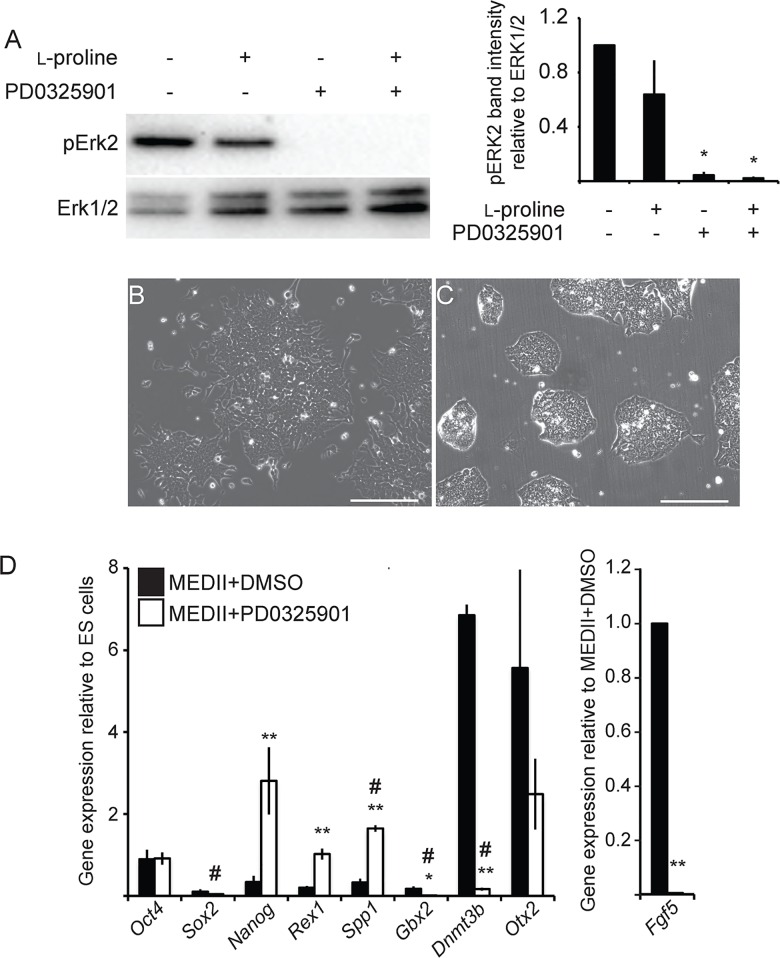
Inhibition of MEK1 prevents the formation of EPL cells in response to MEDII. A. mES cells were pre-treated with 1 μM PD0325901 for 60 minutes. 200 μM l-proline was added, as denoted, and the cells incubated for a further 60 minutes. Cells were collected and analysed by western blot for the presence of phosphorylated ERK1 or ERK2. Total ERK1/2 was used as a loading control. The intensity of the pERK2 band was measured using Quantity One software (BioRad) and represented as a proportion of total ERK1/2. Error bars represent SEM; n = 4; * *p* ≤ 0.05 when compared to mES cells. B-C. mES cells were cultured in MEDII- and DMSO-containing medium (B) and MEDII- and 1μM PD0325901-contianing medium (C) for 3 days. Scale bar = 200 μm. D. MEDII- and DMSO-containing medium (■) and MEDII- and 1μM PD0325901-contianing medium (□) for 3 days. RNA from these cells was analyzed for expression of *Oct4*, *Sox2*, *Nanog*, *Rex1*, *Spp1*, *Gbx2*, *Dnmt3b*, *Otx2* and *Fgf5* by real-time PCR. Expression was normalized to *β-actin* and expressed relative to mES cells (*Fgf5* has been expressed relative to MEDII + DMSO). Error bars represent SEM; n = 3. mES cells + MEDII + PD0325901 were compared to mES cells + MEDII + DMSO (** p ≤ 0.05) or mES cells (# p ≤ 0.05).

EPL cells are routinely formed from primed mES cells in culture [13, 20]. The inhibition of ERK activity in mES cells promotes the transition of primed mES cells to the naive state [9, 11]. The ability of MEDII to induce EPL cells from a mES cell population in which MEK1 signalling had been inhibited, and therefore lacking phosphorylated ERK1/2, was tested. Phosphorylated (p)ERK2 was detected in ES cells; phosphorylation was lost in cells cultured with the MEK inhibitor PD0325901 [30](Table 1; **Fig** 1A). Addition of PD0325901 to ES cells in conjunction with MEDII or l-proline prevented the adoption of EPL cell-like colony morphology (**Fig** 1B and 1C; data not shown). The cells were analysed by qPCR for the expression of markers of pluripotency (*Oct4*, *Sox2* and *Nanog*), the early epiblast of the ICM/ES cells (*Rex1*, *Spp1* and *Gbx2*) and the primitive ectoderm/EPL cells (*Dnmt3b*, *Otx2* and *Fgf5; Fgf5* was only examined in cells cultured in MEDII [20]). Cells cultured in MEDII + PD0325901 failed to up regulated *Fgf5* and *Dnmt3b* expression and maintained *Nanog*, *Rex1* and *Spp1* expression when compared to cells cultured in MEDII, consistent with maintenance of an ES cell-like state in the absence of MEK activity (**Fig** 1D).

**Table 1:** Summary of inhibitors used in this study.

Compound	Chemical name	[μM]	Type of inhibition	Specificity	References^1^
PD0325901	N-[(2R)-2,3-dihydroxypropoxy]-3,4-difluoro-2-[(2-fluoro-4-iodophenyl)amino]-benzamide	1	Non-ATP competitive binding inhibition	MEK1^2^^,^^4^	[9, 11, 31–33]
PP2	Pyrazolo-pyrimidine 4-amino-5-(4-chlorophenyl)-7-(t-butyl)pyrazolo[3,4-d]pyrimidine	10	ATP competitive binding inhibition	Src^4^, p56^Lck,^^4^, p59^FynT,^^4^, Hck^4^, EGFR^5^, RIP2^5^, CK1δ^5^ and GAK^5^.^3^	[34–39]
SB203580	4-(4´-fluorophenyl)-2-(4´-methylsulfinylphenyl)-5-(4´-pyridyl)-imidazole	10	ATP competitive binding inhibition	p38α MAPK^4^, p38β MAPK^4^ and CK1δ^5^.	[31–33, 40–44]
SU6656	2-oxo-3-(4,5,6,7-tetrahydro-1 *H*-indol-2-ylmethylene)-2,3-dihydro-1*H*-indole-5-sulfonic acid dimethylamide		ATP competitive binding inhibition	MAPKAP-K1a^4^, AMPK^4^, PHK^5^, p56^Lck,^^4^ and DYRK1A^4^.	[34, 45, 46];

^1^ Selected references, prioritized to include those reporting on role of inhibitor in ES cell renewal and differentiation.

^2^ Inhibitor is reported to have activity on MEK1 but not on ERK1, ERK2, p38αMAPK or p38βMAPK [34].

^3^ PP2 has been shown to have a modest impact on the activity of p38α MAPK, p38β MAPK [34].

^4^ Detected in ES cells.

^5^ Not reported in ES cells.

### EPL cell formation requires signaling through SRC family kinases

**Fig 2:**
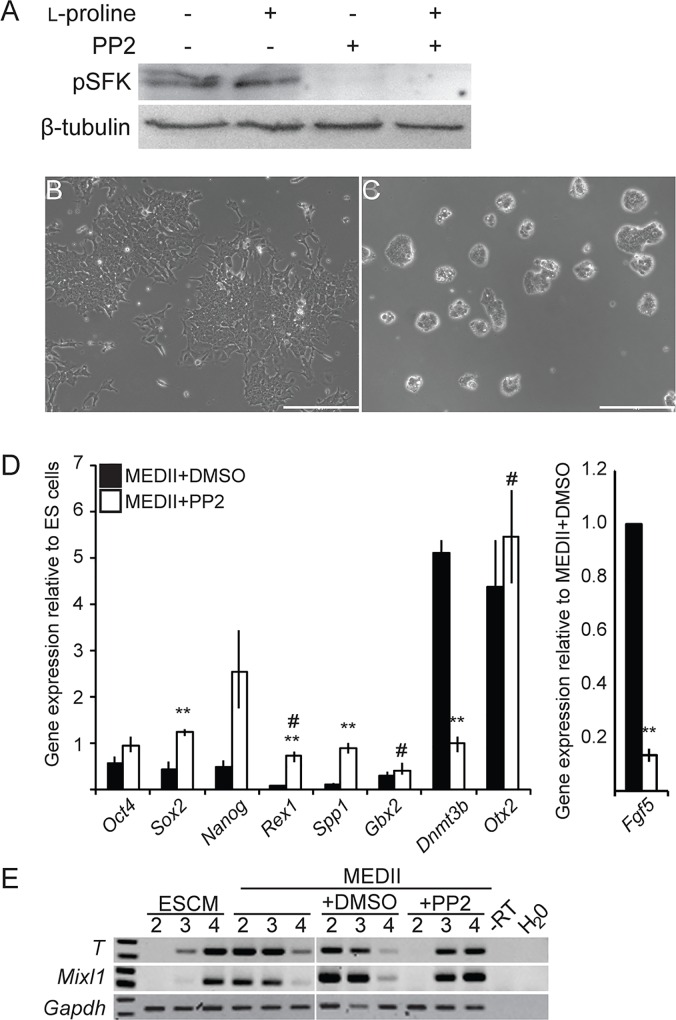
**Src Family Kinases are required for the formation of EPL cells in response to MEDII A.** mES cells were cultured with or without l-proline and PP2, as denoted. Total protein was extracted after 24 hours and analysed by western blot for the presence of phosphorylation of Src family proteins (pSFK). β-tubulin was used to normalise for protein loaded. **B, C.** mES cells were cultured in medium supplemented with MEDII and DMSO (A) or MEDII and 10 μM PP2 (B) for 3 days. Scale bar = 200 μm. **D.** mES cells were cultured in medium supplemented with MEDII and DMSO (■) or MEDII and 10 μM PP2 (□), for 3 days. RNA from these cells was analyzed for transcripts of *Oct4*, *Sox2*, *Nanog*, *Rex1*, *Spp1*, *Gbx2*, *Dnmt3b*, *Otx2* and *Fgf5* by real-time PCR. Expression was normalized to *β-actin* and expressed relative to ES cells (*Fgf5* has been expressed relative to MEDII + DMSO). Error bars represent SEM; n = 3. ES cells in MEDII + PP2 were compared to cells cultured in MEDII + DMSO (** p ≤ 0.05) or ES cells (# p ≤ 0.05). **E.** mES cells were cultured in ES cell medium supplemented with MEDII, DMSO or MEDII and 10 μM PP2 for 3 days and formed into EBs. EBs were collected on days 2, 3 and 4, RNA was isolated and analyzed for expression of *T*, *Mixl1* and *Gapdh* by RT-PCR. n = 3.

There are nine members of the Src family kinases, seven of which have been shown to be expressed in mES cells; c-Src, p56^Lck^, p59^FynT^, Hck, Lyn, Fgr and Yes [30, 38, 47](JK, EW and JR, personal communication), and c-Src activity is required for the formation of a primitive ectoderm-like cell from mES cells in culture [38, 39]. PP2, at a concentration of 10 μM, was used to inhibit Src, p56^Lck^, p59^FynT^ and Hck in mES cells (Table 1). At this concentration the phosphorylation of Src family members was reduced (**Fig** 2A) and cells did not adopt an EPL cell colony morphology in response to MEDII (S1 **Fig**). The addition of PP2 reduced colony size, which was likely a consequence of the inhibition of pluripotent cell proliferation (S1 **Fig**). The addition of PP2 maintained or increased the expression of pluripotent and mES cell markers, with the exception of *Gbx2*, in mES cells cultured in MEDII- or l-proline-containing medium, and reduced the expression of *Dnmt3b* and *Fgf5*, but not *Otx2*, (**Fig** 2D and S2 **Fig**).

When differentiated as embryoid bodies (EBs), EPL cells increase expression of differentiation markers earlier than mES cells [20, 24]. mES cells and EPL cells were aggregated to form EBs and analysed on days 2, 3 and 4 for the expression of markers of the primitive streak (*T*, *Mixl1*, *Fgf8*, *Wnt3* and *Tgfβ1*) and nascent mesoderm (*BMP4*) by RT-PCR (**Fig** 2D). Differentiation markers were up regulated approximately 48 hours earlier in EBs derived from EPL cells when compared to those derived from mES cells. EBs derived from mES cells cultured in MEDII supplemented with PP2 up regulated differentiation markers approximately 24 hours earlier than mES cell-derived EBs (**Fig** 2E, S1 **Fig**). The advancement of differentiated gene expression in EBs derived from cells cultured in l-proline has always been much less than in EBs derived from EPL cells [20]. Despite this, the addition of PP2 to the cells prior to the formation of EBs did impact differentiation kinetics, with a delay in the expression of the differentiation marker *T* (S2 **Fig**).

### p38 MAP Kinase in EPL cell formation

**Fig 3:**
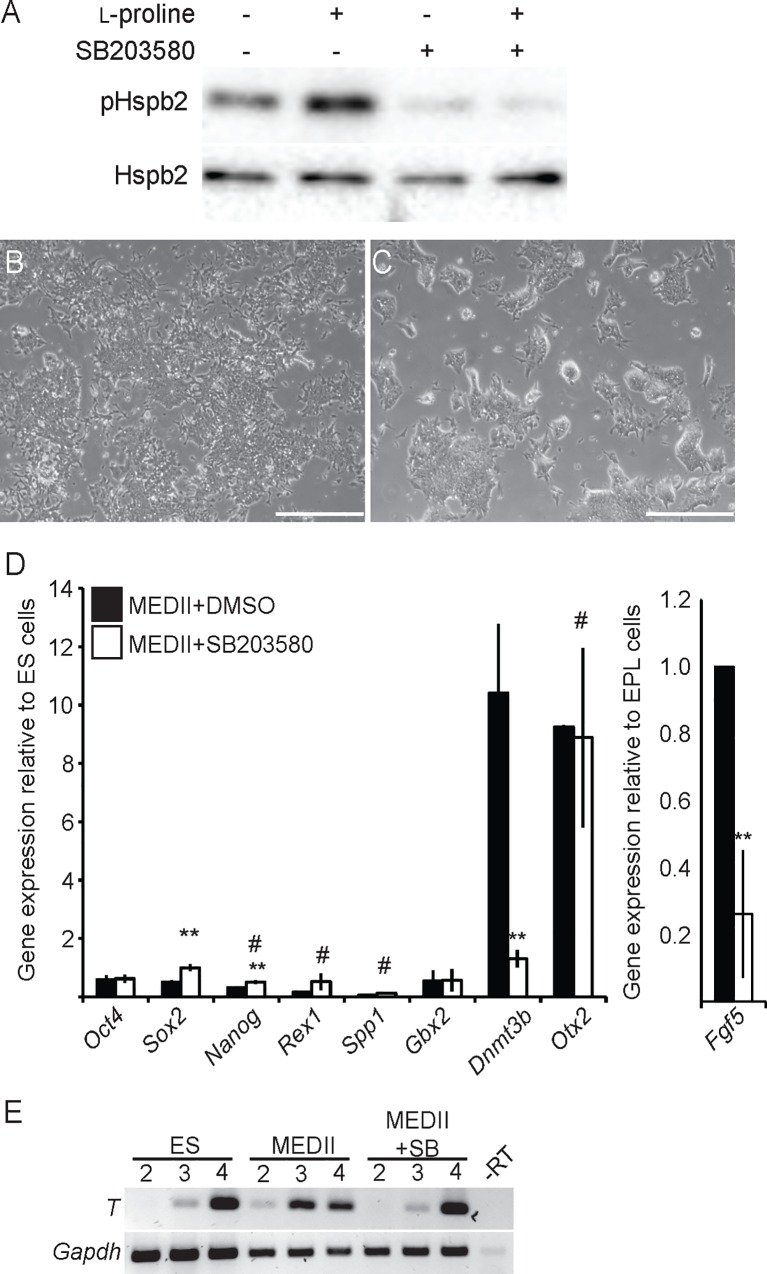
The role of p38 MAPK in the formation of EPL cells in response to MEDII. **A.** mES cells were pre-treated with 10 μM SB203580 for 60 minutes. 200 μM l-proline was added, as denoted and the cells incubated for a further 60 minutes. Cells were collected and analysed by western blot for the presence of phosphorylated pHspb2. Total Hspb2 was used as a loading control. **B, C.** mES cells were cultured medium supplemented with MEDII and DMSO (A) or MEDII and 10 μM SB203580 for 3 days. Scale bar = 200 μm. **D.** mES cells were cultured in medium supplemented with MEDII and DMSO (■) or MEDII and 10 μM SB203580 (□) for 3 days. RNA from these cells was analyzed for expression of *Oct4*, *Sox2*, *Nanog Rex1*, *Spp1*, *Gbx2*, *Dnmt3b*, *Otx2* and *Fgf5* by real-time PCR. Expression was normalized to *β-actin* and expressed relative to mES cells (*Fgf5* has been expressed relative to MEDII + DMSO). Error bars represent SEM; n = 4. mES cells + MEDII + SB203580 were compared to mES cells + MEDII + DMSO (** p ≤ 0.05) or mES cells (# p ≤ 0.05). **E.** mES cells were cultured in ES cell medium supplemented with MEDII, DMSO or MEDII and 10 μM 10 μM SB203580 for 3 days and formed into EBs. EBs were collected on days 2, 3 and 4, RNA was isolated and analyzed for expression of *T* and *Gapdh* by RT-PCR. n = 3.

A kinome screen of mES cells exposed to l-proline showed increased phosphorylation of p38α MAPK, p38β MAPK and Heat shock protein 2 (Hspb2, also known as Hsp27) (S3A **Fig**), suggesting that the p38 MAPK pathway could be activated [48, 49]. MAP kinase pathways have been implicated in early mES cell differentiation decisions and the formation of the germ lineages in culture [31–33, 40–44]. Western blot showed the presence of pHspb2 in mES cells and mES cell treated with l-proline (**Fig** 3A). Phosphorylated Hspb2 levels were significantly reduced in by the inhibitor SB203580 (**Fig** 3A).

The addition of 10 μM SB203580 during EPL cell formation resulted in subtle changes in colony morphology across the population, with many colonies having a more rounded appearance and refractive edges, characteristics of mES cell colonies and not EPL cell colonies (**Fig** 3B and 3C; data not shown). The expression of *Dnmt3b* and *Fgf5*, but not *Otx2*, was reduced with the addition of SB203580 to cells cultured in MEDII-containing or l-proline-containing medium (**Fig** 3D and S2 **Fig**). These data suggest that p38 MAPK was required for the increased expression of these genes. The expression of *Rex1*, *Spp1* and *Gbx2* was generally unaffected by the addition of SB203580 and was not restored to levels comparable those in untreated mES cells. mES cells cultured in MEDII- or l-proline-containing medium and SB203580 were differentiated as EBs and the expression of *T* analysed (**Fig** 3E and S2 **Fig**). SB203580 prevented the acquisition of EPL cell differentiation kinetics.

mES cells that lack the p38α MAPK isoform have been established [50]. *p38α*^*-/-*^cells showed equivalent expression of *Nanog*, *Rex1* and *Spp1* when compared to the parental line, but higher expression of *Gbx2* and significantly lower basal expression of *Dnmt3b*, *Otx2* and *Fgf5* (S3B **Fig**). The addition of MEDII to *p38α*^*-/-*^ mES cells resulted in increased expression of the primitive ectoderm markers. These cells, however, failed to down-regulation the ES cell markers (S3C **Fig**). These data suggest that the loss of *p38α* affects the formation of EPL cells from mES cells but did not recapitulate the addition of SB203580.

### The role of signaling pathways in the maintenance of EPL cells in culture

**Fig 4:**
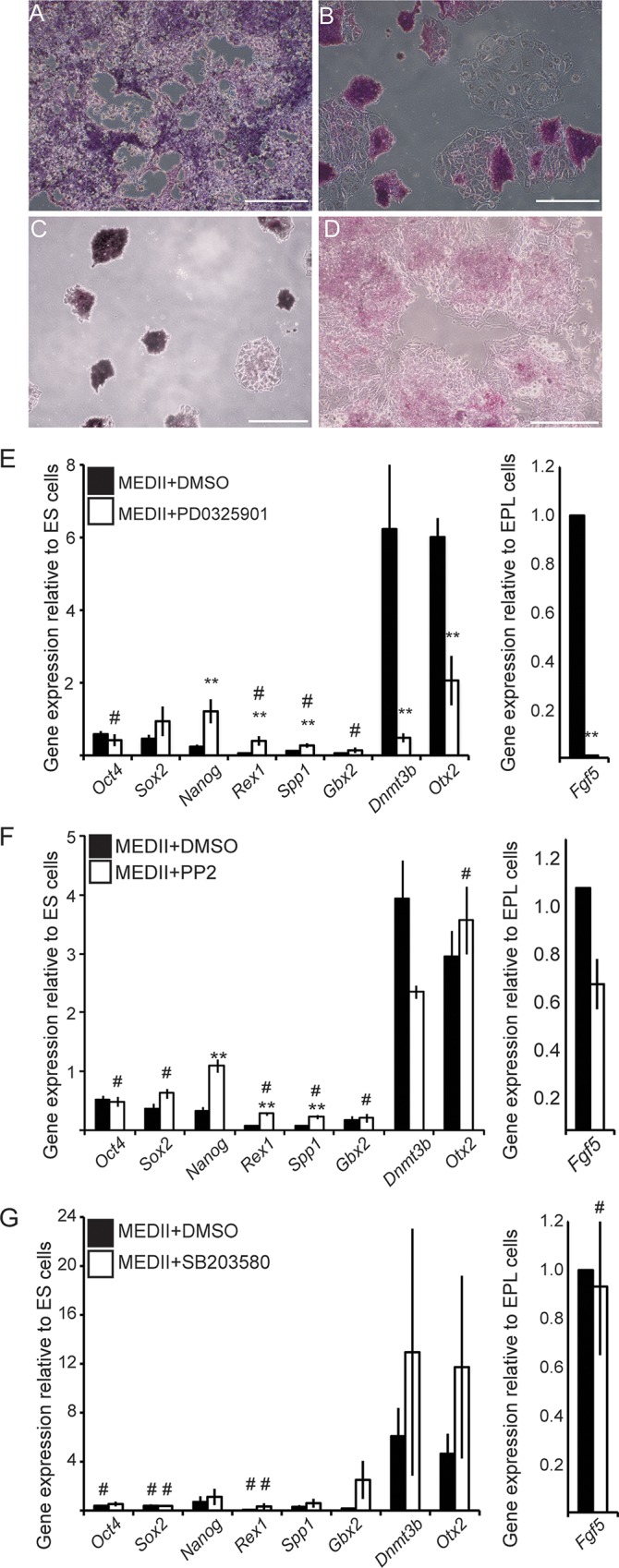
The role of ERK1/2, Src Family Kinases and p38 MAPK in the maintenance of EPL cells. **A-D.** Photomicrographs of mES cells cultured in MEDII for 2 days and subsequently in MEDII containing medium supplemented with DMSO (A), 1 μM PD0325901 (B) 10 μM PP2 (C) and 10 μM SB203580 (D). Cells were stained for alkaline phosphatase activity (red/purple stain). Scale bar = 200 μm. **E, F.** mES cells were cultured in MEDII for 2 days and subsequently in MEDII containing medium supplemented with DMSO (■), DMSO with 1 μM PD0325901 (E, □), DMSO with 10 μM PP2 (F, □) or DMSO with 10 μM SB203580 (G, □). RNA from these cells was analyzed for expression of *Oct4*, *Sox2*, *Nanog*, *Rex1*, *Spp1*, *Gbx2*, *Dnmt3b* and *Otx2* by real-time PCR. Expression was normalized to *β-actin*. Error bars represent SEM; n = 3. EPL cells cultured in MEDII with inhibitor were compared to EPL cells in MEDII with DMSO, ** *p* ≤ 0.05, or mES cells, # *p*≤ 0.05.

Signaling through the ERK2, Src family kinase and p38 MAPK all play a role in the formation of EPL cells from mES cells. Signaling inhibitors were added to extant EPL cells to assess the roles these pathways may play in EPL cell maintenance. Inhibition of MEK1/ERK2 in EPL cells resulted in a mixed population of pluripotent and differentiated cell colonies, with alkaline phosphatase positive cell colonies, adopting a compact, 3-dimensional structure rather than an epithelial structure (**Fig** 4A and 4B). The population showed a significant up regulation of *Nanog*, *Rex1* and *Spp1* expression, maintenance of *Oct4* and *Sox2* expression and a reduction in EPL cell marker expression when compared to EPL cells (**Fig** 4E). The addition of PP2 to EPL cells also disrupted colony morphology, with a number, but not all, colonies within the culture rounding up (**Fig** 4C); these colonies were positive for alkaline phosphatase activity. These cells expressed significantly more *Nanog* and *Sox2* compared to EPL cells. Increased expression of *Rex1* and *Spp1*, and decreased expression of *Dnmt3b* and *Fgf5*, in these cells was consistent with the cells acquiring a more ES cell-like gene expression profile (**Fig** 4F). These changes did not all reach parity with ES cell gene expression (data not shown). The expression of *Gbx2* and *Otx2* was unaffected by the addition of PP2. EPL cells in which p38 MAPK had been inhibited appeared to be more differentiated than controls and there were qualitatively fewer alkaline phosphatase cells (**Fig** 4D). Gene expression analysis detected no significant differences in the expression of the pluripotent, ES cell or EPL cell markers (**Fig** 4G), suggesting that an EPL cell gene expression profile was maintained. Despite the appearance of more differentiated cells within this population, gene expression analysis could not detect a role for p38 MAPK in EPL cell maintenance.

### ROS-signalling and EPL cell formation

**Fig 5:**
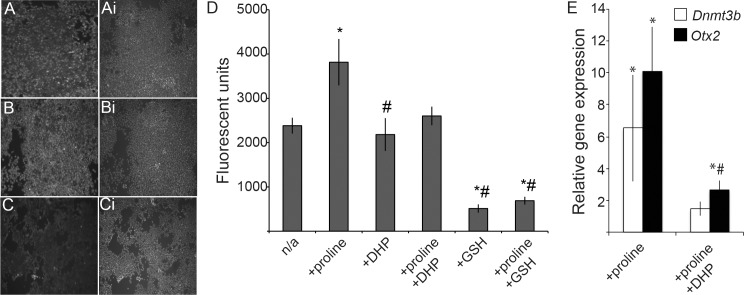
The addition of l-proline to ES cells increases ROS. **A-C**. MitoSox Red staining of mES incubated with 200 μM l-proline (B) or l-proline and ascorbic acid (C) and compared to untreated cells (A). Hoechst staining of the same fields of view are shown in Ai, Bi and Ci. D. ROS levels were measured as fluorescence using ROS-Glo™ H_2_O_2_ Assay (Promega) in cells that had been incubated with l-proline, 150 μM DHP or 1 mM GSH, as denoted. Results are shown as arbitrary fluorescent units. n = 4, *p≤0.05 when compared with mES cells. # p≤0.05 when compared with mES cells incubated with l-proline. E. The expression of *Dnmt3b* and *Otx2* was analysed in mES cells cultured in medium supplemented 200 μM l-proline or 200 μM l-proline and 150 μM DHP. Expression was normalized to β-actin and expressed relative to mES cells. Error bars represent SEM; n = 4. Comparisons were made to mES cells (*p ≤ 0.05) or mES cells cultured with 200 μM l-proline (# p ≤ 0.05).

Several lines of evidence suggest that c-SRC can be activated by increased intracellular ROS [16, 51]. Likewise, others have implicated ROS in the activation of p38 MAPK (for example [52–54]). Metabolism of l-proline by proline dehydrogenase within the cell can result in increased intracellular ROS levels [55], providing a potential link between l-proline uptake and signaling pathway activation. mES cells with and without l-proline were assayed for steady state levels of intracellular ROS qualitatively and quantitatively (**Fig** 5). Qualitatively, a population of ES cells comprised a mix of ROS-bright and ROS-dull cells (**Fig** 5A); this was reflected in a basal level of biochemically detected ROS within the population (**Fig** 5D). The addition of l-proline resulted in an increase in the number of ROS-bright cells in the population and a concomitant increase in the levels of ROS detected (**Fig** 5B and 5D). The PRODH inhibitor 3,4-dehydro-L-proline (DHP) reduced the levels of ROS in l-proline-treated cells to basal levels, but had no impact on ROS levels in untreated mES cells. Addition of an antioxidant, ascorbic acid (**Fig** 5C) or glutathione (GSH; **Fig** 5D), abolished ROS-bright cells in the population and reduced the levels of ROS to below basal (mES cell) levels. Finally, inhibiting l-proline metabolism with DHP reduced the expression of *Dnmt3b* and *Otx2* in l-proline-treated cells (**Fig** 5E), suggesting a requirement for metabolism in the regulation of EPL cell gene expression.

## Discussion

### EPL cell inductive factors act on a primed ES cell population

mES cells can be cultured in the naïve or the primed state. The naive pluripotent cell state is achieved by preventing signaling through ERK1/2 in combination with a GSK3ß inhibitor, and thereby maintaining cells in an intrinsically self-maintaining state regulated by Nanog and shielded from differentiation signals [9, 11]. With increasing ERK signaling mES cells become primed, or able to respond to differentiation signals [31], while deletion of Erk2-/- biases cells towards self-renewal [56]. Inhibition of ERK2 signaling prevented the formation of EPL cells in response to l-proline or MEDII, suggesting that active ERK2, and the concomitant primed mES cell state, was required for EPL cell formation. Inhibition of Src activity can replace the inhibition of ERK signaling in the maintenance of naïve mES cells [57, 58], and c-Src inhibition has been shown to promote the formation of naïve human pluripotent cells [59]. The ability of PP2 to inhibit the formation of EPL cells from ES cells in response to l-proline or MEDII is consistent with a requirement for a primed mES cell substrate in primitive ectoderm formation.

### Acquisition and maintenance of the EPL cell state requires the activity of multiple intracellular pathways

Src family kinase signaling was required for the maintenance of the EPL cell state in culture, and suggests a role for this pathway beyond facilitating mES cell priming. Several reports have noted the functional similarity between human (h)ES cells and primitive ectoderm-like cell lines from mouse, EPL cells and EpiSCs [26, 60, 61]. Pluripotency in hES cells is maintained, in part, by fibroblast growth factor-2 (FGF-2). Analysis of the pathways activated by FGF signalling in hES cells demonstrated tyrosine phosphorylation of Src family kinases and Src substrates [62]. This was interpreted as indicating a role for Src kinase activity in the maintenance of hES cell pluripotency by FGF-2, and is consistent with the role of Src family kinase activity in maintaining the primitive ectoderm-like identity of EPL cells in culture.

The formation of EPL cells from mES cells, like the formation of primitive ectoderm from pluripotent cells of the blastocyst, is accompanied by epithelialisation of the pluripotent cells. PP2 prevented this change in colony morphology and promoted growth in compact colonies; others have seen this effect [63]. This effect has also been seen in non-pluripotent cells, in which PP2 inhibited cell migration, and also, surprisingly, in fibroblasts deficient in c-Src, Yes and Fyn, suggesting that migration in these cells depended on a non-Src family kinase mechanism that is affected by PP2 [63]. Consistent with this, the Src family inhibitor SU6656 did not inhibit the epithelialisation of mES cells cultured in MEDII (Data not shown). It is yet to be determined if the mechanisms that regulate colony morphology changes seen in pluripotent cell lineage progression are equivalent to those that regulate migration in somatic cells.

Treatment of mES cells with l-proline resulted in increased p38 MAPK phosphorylation. Inhibition of p38 MAPK activity during EPL cell formation impacted the change in colony morphology, prevented the up regulation of primitive ectoderm markers *Dnmt3b* and *Fgf5* and blocked the change in differentiation kinetics that accompanies EPL cell formation, suggesting a role for p38 MAPK in the establishment of EPL cells. Inhibiting signaling through p38 MAPK did not, however, restore the expression of pluripotent / ES cell markers. The deletion of *p38α* resulted reduced basal expression of primitive ectoderm markers when compared to WT cells; expression of these markers increased with the addition of MEDII but there was no accompanying alteration in differentiation kinetics (data not shown). Previous reports showed that knockdown of p38δ, the second highest p38 MAPK isoform found in ES cells, in *p38α*^*-/-*^ ES cells did not affect ES cell differentiation [64]. Although the loss of *p38α* did prevent aspects of EPL cell formation, *p38α*^*-/-*^ mES cells do not phenocopy mES cells cultured in a p38 MAPK inhibitor, suggesting that in the absence of other *p38α* factors, including *p38β*, may regulate EPL cell marker gene expression.

### Multiple pathways regulate pluripotent lineage progression

MES cells in culture do not grow as a homogenous population but exist in a metastable state. Heterogeneity has been revealed by the non-uniform expression of *Zfp42*, *Dppa3*, *Nanog*, *Pecam1* and *Otx2* in cells expressing *Oct4* [65–70]. These genes mark interchangeable pluripotent cell states corresponding to an ICM-like, *Otx2*-low state and a later pluripotent cell state, marked by higher *Otx2* expression, that coexist and ensure the self-renewal and perpetuation of pluripotency and a susceptibility to differentiation factors [10]. *Otx2* has been shown to be intrinsic to the metastable state; overexpression of *Otx2* resulted in a population of cells representative of late epiblast of the post-implantation embryo and EpiSC, while *Otx2*^*-/-*^ mES cells are highly enriched for *Nanog*-expressing cells and predisposed to self-renewal [69]. Otx2 has also been shown to be required for the transition of mES cells from the ICM-like state and later, primed, pluripotent cell states [71]. *Otx2* has been used here as a marker of EPL cells. Inhibition of neither Src family kinases, p38 MAPK, nor MEK1/ERK2 affected the regulation of *Otx2* expression during the formation of EPL cells, although the loss of ERK1/2 signalling in formed EPL cells did reduce the expression of *Otx2* (S4 **Fig**). The inability to prevent *Otx2* expression when mES cells are exposed to l-proline or MEDII may underlie the failure of these inhibitors to prevent all aspects of differentiation and to maintain an mES cell in culture. Several transcriptional modules have been identified in the regulation of mES cells pluripotency, each potentially regulating different gene cohorts and regulated by different upstream triggers [72–76]. It is likely that Src family kinases, p38 MAPK and ERK2 signaling regulate some, but not all, of these transcriptional modules, resulting in the incomplete inhibition of EPL cell formation and maintenance of *Otx2* expression.

### Multiple pluripotent cell states in culture

**Fig 6:**
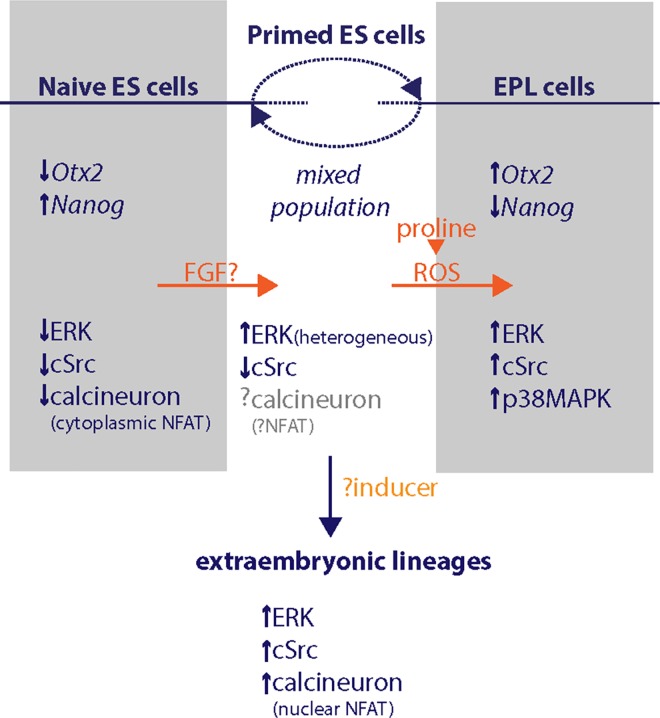
The regulation of progression of the pluripotent lineage in culture. The cell states represented in vitro, naïve mES cells, primed mES cells and EPL cells have been aligned with the expression of Nanog and Otx2 and with their deduced intracellular signaling activity. Inducers of lineage progression are shown in orange; Calcineurin exerts its effects through dephosphorylation of NFAT and promotes NFAT translocation to the nucleus.

The regulation of pluripotent lineage progression is not well understood, but these findings, coupled with the findings of others, can be integrated into a model of the process (**Fig** 6). In culture, mES cells in the naïve state are hypothesized to represent the earlier pluripotent cells of the lineage [12, 77]. In culture, these cells are maintained by supressing GSK3β signaling in combination with the inhibition of ERK1/2 signaling (through the inhibition of MEK1), Src family kinases or calcineurin [9, 11, 57, 58]. It is thought that calcineurin signaling collaborates with ERK signaling to activate Src, which in turn drives the priming of the cells for differentiation [58]; inhibition of any of these signalling components will prevent Src activation. In primed mES cells ERK activity is present. The absence of calcineurin signaling in these cells [58] will likely prevent the up regulation and activation of c-Src in response to ERK1/2 signaling.

The transition of mES cells to EPL cells involves the activation of Src family kinases and p38 MAPK and occurs in response to l-proline. The addition of l-proline to primed mES cells increases ROS within the cell population. The enzyme central to proline metabolism in the cell, proline dehydrogenase (PRODH or POX), converts l-proline to Δ^1^-pyrroline-5-carboxylate (P5C), with the concomitant transfer of electrons to oxygen and generation of ROS [78, 79]. ROS increases in mES cells were prevented by inhibition of PRODH, and EPL cell morphology could be prevented by ROS scavengers and PRODH inhibition [21], suggesting a role for proline metabolism in mES cell differentiation. c-Src and p38 MAPK can be activated by increased intracellular ROS [51, 53, 54, 80], providing a pathway for the control of differentiation by l-proline. The action of MEDII and l-proline, through ROS induction, does not appear to be able to overcome the suppression of Src activation that can be achieved when MEK1 is inhibited.

The question arises to the relevance of calcineurin or l-proline in the regulation of Src activity within ES cells. The combination of ERK and calcineurin resulted in differentiation of mES cells to cells of the extraembryonic lineages, primarily trophectoderm and endoderm [58], whereas the combination of ERK and l-proline results in the formation of EPL cells and subsequently cells of the germ lineages [15, 24, 25, 81]. These likely represent different pathways to activate c-Src that achieve alternate cellular outcomes, potentially through the differential regulation of p38 MAPK, and place Src family kinase signaling as pivotal in ES cell differentiation.

## Materials and Methods

### Cell culture

D3 mouse mES cell line [82] was maintained as described previously in Rathjen and Rathjen, 2003 [83]. EPL cells were formed by culturing mES cells in medium supplemented with 50% MEDII, or medium supplemented with LIF and 200 μM l-proline (Sigma-Aldrich) [20, 83]. Chemical inhibitors (PP2 (Calbiochem), SB203580 (Tocris Bioscience), SU6656 (Sigma Aldrich) and PD0325901 (Selleck Chemicals)) were added every 24 hours or as described in the text. Inhibition of SRC family kinase phosphorylation by PP2 was still apparent at 24 hours (**Fig** 1G), suggesting this inhibitor was stable in these culture conditions. Inhibition p38 MAPK by SB203580 was clear after 60 minutes (**Fig** 3A) but was less predictable at 24 hours (data not shown), suggesting activity remained after extended culture but some potency had been lost. Inhibition of ERK2 phosphorylation by PD0325901 was obvious after 60 minutes (**Fig** 5A) but was not detected at 24 hours (data not shown), suggesting this inhibitor was not stable in culture over this time. MES cells were seeded at a density of 2.5 x 10^4^ cells / cm^2^ onto tissue culture-treated plasticware (Nunc^TM^), pretreated with 0.2% fetal porcine gelatin (Sigma-Aldrich). Cells were cultured for 3 days (in 50% MEDII) or 4 days (with 200 μM l-proline) with daily medium replenishment. Chemical inhibitors were suspended in dimethyl sulfoxide (DMSO) (Sigma-Aldrich) and added to cells as described in the text. For EPL cell maintenance experiments mES cells were cultured in medium supplemented with 50% MEDII for 2 days, followed by the addition of chemical inhibitors to the medium for a further 2 days, as described in text. Alkaline phosphatase activity was performed according to manufacturer’s instructions using an alkaline phosphatase detection kit (Sigma-Aldrich; catalogue # 86C) with modifications to allow staining in tissue culture plasticware [13]. Images were taken on an Olympus IX51 with an F-view II digital camera (Olympus) or UC-30 digital camera. Embryoid bodies (EBs) were formed from mES cells that had been cultured for 3 days (in 50% MEDII) or 4 days (with 200 μM l-proline) as described previously [83]; chemical inhibitors were added as described in text.

### Gene expression analysis

Reverse Transcriptase-PCR (RT-PCR): Total RNA was isolated from cells using TRIzol® reagent (Invitrogen^TM^) as per the manufacturer’s instructions and DNaseI treated (Ambion). cDNA synthesis was performed using M-MLV Reverse Transcriptase (Promega). PCR reactions, containing GoTaq® Green Master Mix (Promega), 200 nM each of forward and reverse primers and 1 μl of cDNA were initially heated to 95°C for 2 minutes and then cycled through 95°C for 30 seconds, 60°C for 30 seconds and 72°C for 30 seconds on a MJ Research or Bio-rad thermocycler. Primer sequences, the number of cycles used for each primer pair and the size of the expected amplicon are listed in S1 Table.

### Quantitative Real-time PCR (qPCR)

RNA was isolated, DNaseI treated and cDNA was synthesized as for RT-PCR. Reactions, consisting of cDNA, HOT FirePol EvaGreen Mix (Integrated Sciences) with 200 nM each of forward and reverse primers, were amplified using MJ research thermocycler with a Chromo4 Continuous Fluorescence Detection System (MJ Research) or Via^TM^ 7 real time PCR system. The samples were initially heated to 95°C for 15 minutes followed by 40 cycles of 95°C for 15 s, 60°C for 15 s and 72°C for 30 s. The cycle threshold (Ct) values for *β-actin* were normalized across samples and the raw Ct values were analyzed using Q-Gene software package [84]. Primer sequences are listed in S1 Table.

### Western Blot

Total cellular protein was extracted from mES cells with 200 μl Laemmli sample buffer (Bio-rad) containing β-mercaptoethanol (Sigma-Aldrich), protease inhibitor (#P-8350; Sigma-Aldrich) and phosphatase inhibitor, as per manufacturer’s instructions. Protein extract was incubated at 100°C for 10 minutes and separated using a 10% SDS polyacrylamide gel. Standard immunoblot procedures were followed and proteins were visualized using the Immun-Star^TM^ WesternC^TM^ Chemiluminescence Kit (Bio-Rad) and a Bio-Rad ChemiDoc^TM^ XRS as per the manufacturer’s instructions. Antibodies, antibody suppliers and dilutions used are stated in S1 Table. Band intensities were estimated using Quantity One software (Bio-Rad).

### Kinome array

mES cells were transferred to DMEM + 0.1% FCS (Life technologies) for 4 hours prior to setting up the assay. At time 0, cells were mechanically removed from the plastic and aliquots of approximately 1 x 10^7^ cells were treated with LIF (5 minutes), 200 μM l-proline (5 and 15 minutes) or maintained in DMEM + 0.1% FCS (15 minutes). Cells were lysed and lysates applied to a Proteome Profiler^TM^ Human Phospo-MAPK Array (R&D Systems, catalogue # ARY002B) as per the manufacturer’s instructions. Signals were developed using enhanced chemiluminescence (GE Lifescience) and quantified using QuantityOne (Bio-Rad).

### BrdU Assay

mES cells were cultured with 5’ Bromo 2’ deoxyuridine (BrdU) (10 μM; Sigma Aldrich) for 2 hours before cells were washed and reduced to a single cell suspension. Cells were fixed in ice-cold 70% ethanol, permeabilised in 1 M HCl, and BrdU incorporation detected using an α-BrdU monoclonal antibody (Bioclone Australia) in combination with a rabbit α-mouse IgG Alexa Fluor 488 secondary antibody (Invitrogen). Cells that had incorporated BrdU were quantified by flow cytommetry.

### ROS analysis

#### Live cell imaging

mES cells were seeded at 2.5 x 10^4^ cells per well into 96 well black imaging plates (BD Falcon, 31053) and cultured for 4 days. L-proline (200 μM) was added on day 3, and ascorbic acid (200 μM) was added 4 hours prior to imaging. 30 minutes prior to imaging, MitoSOX™ Red mitochondrial superoxide indicator (5 μM, Invitrogen, M36008) and Hoechst (Life Technologies, H3570) were added as per the manufacturer’s specifications. Cells were washed in live cell imaging buffer (DMEM without Phenol Red (Gibco, 31053), 10% Foetal calf serum (Life Technologies), 0.1mM β-mercaptoethanol (Sigma)) and imaged using a Leica DMIRB inverted fluorescent microscope (Leica Microsystems), connected to a Hamamatsu ORCA-ER digital camera. NIS Elements D software (version 4.0, Nikon®) was used to view the images and control exposure times.

#### Biochemical analysis of ROS

ROS production was quantified using the ROS-Glo™ H_2_0_2_ Assay (Promega, G8820). MES cells were seeded at 1 x 10^6^ cells per well into a 96 well white imaging plates (BD Falcon, 351130) and allowed to adhere overnight. 200 μM l-proline was added at the time of seeding as denoted in the text. 4 hours prior to performing the assay, DHP (150 μM; Sigma Aldrich) or 1 mM GSH (Sigma-Aldrich, PHR1359) were. The H_2_O_2_ substrate solution was added 1 hour after treatments were added and the plate was incubated at 37°C for an additional 3 hours. Cells were washed with PBS, the ROS-Glo detection solution was added to with PBS to a total volume of 200 μl and the cells were incubated at room temperature for 20 minutes. Luminescence readings were taken using the GloMax® -96 Microplate luminometer (Promega).

### Statistical Analysis

Experiments were analyzed using one-way ANOVA followed by Tukey’s multiple comparison post-hoc test and significance was achieved at p = 0.05 or less with the use of Graphpad Prism.

## Supporting Information

S1 Fig PP2 impacts the colony morphology, proliferation rate and differentiation kinetics of pluripotent cells in cluture. A. ES cells were cultured in ESCM and ESCM + 200 μM L-proline with or without 10 μM PP2, as indicated, for 4 days. Scale bar = 200 μm. B. ES cells were cultured in MEDII + DMSO and MEDII + 5, 10 or 20 μM PP2 for 3 days. Scale bar = 200 μm. Although a clear change in the appearance of colonies could be seen with 5 μM PP2 when compared to MEDII + DMSO, full suppression of EPL cell morphology was seen at 10 and 20 μM. C. ES cells were cultured with 5 and 20 μM PP2 for 5 (■) or 6 (□) days. Cells in S-phase were identified by immunofluorescence for incorporated BrdU followed by flow cytometry. The number of cells incorporating BrdU is shown relative to ES cells. Error bars represent SEM; n = 3. Comparisons were made to untreated ES cells, ** p ≤ 0.01. D. ES cells were cultured in ESCM, MEDII, MEDII + DMSO and MEDII + 10 μM PP2, as indicated, for 3 days and formed into EBs. EBs were collected on days 2, 3 and 4. RNA was isolated and analyzed for expression of *Fgf8*, *Wnt3*, *Tgfβ1*, *Bmp4* and *Gapdh* by RT-PCR; n = 3, a representative image is shown.
(TIF)

S2 Fig The impact of signaling inhibitors on the action of l-proline. **A, B**. ES cells were cultured in medium supplemented with 200 μM l-proline and DMSO (■) or l-proline and 10 μM PP2 (□) (A) or 10 μM SB203580 (□)(B), for 4 days. RNA from these cells was analyzed for transcripts of *Oct4*, *Sox2*, *Nanog*, *Rex1*, *Spp1*, *Gbx2*, *Dnmt3b*, *Otx2* and *Fgf5* by real-time PCR. Expression was normalized to β-actin and expressed relative to ES cells. Error bars represent SEM; n = 3. ES cells in Proline + PP2 were compared to cells cultured in Proline + DMSO (** *p* ≤ 0.05) or ES cells (# *p* ≤ 0.05). **C, D**. ES cells were cultured with l-proline + DMSO, l-proline +10 μM PP2 (C) and L-proline +10 μM SB203580 (D) for 4 days before being formed into embryoid bodies (EBs). EBs were analysed as for the expression of T by RT-PCR. n = 3.
(TIF)

S3 Fig A role for p38 MAPK signaling in EPL cell formation. **A**. ES cells were treated with LIF or 100 μM of l-proline for 5 or 20 minutes, as indicated. Cells were lysed and protein bound to the kinome array and binding quantified. Binding to antibodies specific for pp38α, pp38β, pHsbp2 and MSK2 is shown; n = 2, values have been averaged. **B.** WT ES cell and p38α KO ES cells were cultured in ESCM. RNA was analyzed by qPCR for the expression of *Nanog*, *Rex1*, *Spp1*, *Gbx2*, *Fgf5*, *Dnmt3b* and *Otx2*. Expression was normalized to *Oct4* and expressed relative to WT ES cells. Error bars represent SEM; n = 3. **p ≤ 0.01 when compared to WT ES cells. Loss of p38α decreased expression of primitive ectoderm markers in the ES cell population. C. p38α KO ES cells were cultured in ESCM and MEDII for 3 days to form EPL cells. RNA was analyzed by qPCR for the expression of *Nanog*, *Rex1*, *Spp1*, *Gbx2*, *Fgf5*, *Dnmt3b* and *Otx2* by real-time PCR. Expression was normalized to *Oct4* and expressed relative to p38α KO ES cells. Error bars represent SEM; n = 3. **p ≤ 0.01, *p ≤ 0.05 when compared to KO ES cells.
(TIF)

S4 Fig The effect of inhibiting Src family kinase signaling with SU6656. ES cells were cultured in MEDII + DMSO and MEDII + 1, 2 or 4 μM SU6656 for 3 days. Scale bar = 200 μm.
(TIF)

S1 Table Details of primers and antibodies used in this research. (DOCX)

## References

[1] Chazaud C, Yamanaka Y, Pawson T, Rossant J. Early lineage segregation between epiblast and primitive endoderm in mouse blastocysts through the Grb2-MAPK pathway. Dev Cell. 2006;10(5):615–24. 10.1016/j.devcel.2006.02.020 . 16678776

[2] Pera MF, Tam PP. Extrinsic regulation of pluripotent stem cells. Nature. 2010;465(7299):713–20. 10.1038/nature09228 . 20535200

[3] Rathjen J. The States of Pluripotency: Pluripotent Lineage Development in the Embryo and in the Dish. ISRN Stem Cells. 2014;2014 Article ID 208067. 10.1155/2014/208067

[4] Evans MJ, Kaufman MH. Establishment in culture of pluripotential cells from mouse embryos. Nature. 1981;292(5819):154–6. 10.1038/292154a0 . 7242681

[5] Martin GR. Isolation of a pluripotent cell line from early mouse embryos cultured in medium conditioned by teratocarcinoma stem cells. Proc Natl Acad Sci U S A. 1981;78(12):7634–8. 10.1073/pnas.78.12.7634 6950406 PMC349323

[6] Brons IG, Smithers LE, Trotter MW, Rugg-Gunn P, Sun B, Chuva de Sousa Lopes SM, et al Derivation of pluripotent epiblast stem cells from mammalian embryos. Nature. 2007;448(7150):191–5. 10.1038/nature05950 . 17597762

[7] Tesar PJ, Chenoweth JG, Brook FA, Davies TJ, Evans EP, Mack DL, et al New cell lines from mouse epiblast share defining features with human embryonic stem cells. Nature. 2007;448(7150):196–9. 10.1038/nature05972 . 17597760

[8] Kojima Y, Kaufman-Francis K, Studdert JB, Steiner KA, Power MD, Loebel DA, et al The transcriptional and functional properties of mouse epiblast stem cells resemble the anterior primitive streak. Cell Stem Cell. 2014;14(1):107–20. 10.1016/j.stem.2013.09.014 . 24139757

[9] Ying QL, Wray J, Nichols J, Batlle-Morera L, Doble B, Woodgett J, et al The ground state of embryonic stem cell self-renewal. Nature. 2008;453(7194):519–23. 10.1038/nature06968 . 18497825 PMC5328678

[10] Silva J, Barrandon O, Nichols J, Kawaguchi J, Theunissen TW, Smith A. Promotion of reprogramming to ground state pluripotency by signal inhibition. PLoS Biol. 2008;6(10):e253 10.1371/journal.pbio.0060253 18942890 PMC2570424

[11] Silva J, Nichols J, Theunissen TW, Guo G, van Oosten AL, Barrandon O, et al Nanog is the gateway to the pluripotent ground state. Cell. 2009;138(4):722–37. 10.1016/j.cell.2009.07.039 19703398 PMC3437554

[12] Boroviak T, Loos R, Bertone P, Smith A, Nichols J. The ability of inner-cell-mass cells to self-renew as embryonic stem cells is acquired following epiblast specification. Nat Cell Biol. 2014;16(6):516–28. 10.1038/ncb2965 . 24859004 PMC4878656

[13] Rathjen J, Lake JA, Bettess MD, Washington JM, Chapman G, Rathjen PD. Formation of a primitive ectoderm like cell population, EPL cells, from ES cells in response to biologically derived factors. J Cell Sci. 1999;112 (Pt 5):601–12. . 9973595 10.1242/jcs.112.5.601

[14] Pelton TA, Sharma S, Schulz TC, Rathjen J, Rathjen PD. Transient pluripotent cell populations during primitive ectoderm formation: correlation of in vivo and in vitro pluripotent cell development. J Cell Sci. 2002;115(Pt 2):329–39. . 11839785 10.1242/jcs.115.2.329

[15] Harvey NT, Hughes JN, Lonic A, Yap C, Long C, Rathjen PD, et al Response to BMP4 signalling during ES cell differentiation defines intermediates of the ectoderm lineage. J Cell Sci. 2010;123(Pt 10):1796–804. 10.1242/jcs.047530 . 20427322

[16] Zhang Y, Li TS, Lee ST, Wawrowsky KA, Cheng K, Galang G, et al Dedifferentiation and proliferation of mammalian cardiomyocytes. PloS one. 2010;5(9):e12559 10.1371/journal.pone.0012559 20838637 PMC2933247

[17] Guo G, Yang J, Nichols J, Hall JS, Eyres I, Mansfield W, et al Klf4 reverts developmentally programmed restriction of ground state pluripotency. Development. 2009;136(7):1063–9. 10.1242/dev.030957 19224983 PMC2685927

[18] Hanna J, Markoulaki S, Mitalipova M, Cheng AW, Cassady JP, Staerk J, et al Metastable pluripotent states in NOD-mouse-derived ESCs. Cell Stem Cell. 2009;4(6):513–24. 10.1016/j.stem.2009.04.015 19427283 PMC2714944

[19] Tan BS, Lonic A, Morris MB, Rathjen PD, Rathjen J. The amino acid transporter SNAT2 mediates L-proline-induced differentiation of ES cells. Am J Physiol Cell Physiol. 2011;300(6):C1270–9. 10.1152/ajpcell.00235.2010 . 21346154

[20] Washington JM, Rathjen J, Felquer F, Lonic A, Bettess MD, Hamra N, et al L-Proline induces differentiation of ES cells: a novel role for an amino acid in the regulation of pluripotent cells in culture. Am J Physiol Cell Physiol. 2010;298(5):C982–92. 10.1152/ajpcell.00498.2009 . 20164384

[21] Casalino L, Comes S, Lambazzi G, De Stefano B, Filosa S, De Falco S, et al Control of embryonic stem cell metastability by L-proline catabolism. J Mol Cell Biol. 2011;3(2):108–22. 10.1093/jmcb/mjr001 . 21307025

[22] Comes S, Gagliardi M, Laprano N, Fico A, Cimmino A, Palamidessi A, et al L-Proline Induces a Mesenchymal-like Invasive Program in Embryonic Stem Cells by Remodeling H3K9 and H3K36 Methylation. Stem Cell Reports. 2013;1(4):307–21. 10.1016/j.stemcr.2013.09.001 24319666 PMC3849245

[23] Rathjen J, Rathjen PD. Formation of neural precursor cell populations by differentiation of embryonic stem cells in vitro. ScientificWorldJournal. 2002;2:690–700. 10.1100/tsw.2002.134 . 12805994 PMC6009522

[24] Lake J, Rathjen J, Remiszewski J, Rathjen PD. Reversible programming of pluripotent cell differentiation. J Cell Sci. 2000;113 (Pt 3):555–66. . 10639341 10.1242/jcs.113.3.555

[25] Vassilieva S, Goh HN, Lau KX, Hughes JN, Familari M, Rathjen PD, et al A system to enrich for primitive streak-derivatives, definitive endoderm and mesoderm, from pluripotent cells in culture. PloS one. 2012;7(6):e38645 10.1371/journal.pone.0038645 22701686 PMC3372479

[26] Hughes JN, Dodge N, Rathjen PD, Rathjen J. A novel role for gamma-secretase in the formation of primitive streak-like intermediates from ES cells in culture. Stem Cells. 2009;27(12):2941–51. 10.1002/stem.218 . 19750540

[27] Rodda SJ, Kavanagh SJ, Rathjen J, Rathjen PD. Embryonic stem cell differentiation and the analysis of mammalian development. Int J Dev Biol. 2002;46(4):449–58. . 12141431

[28] Yap C, Goh HN, Familari M, Rathjen PD, Rathjen J. The formation of proximal and distal definitive endoderm populations in culture requires p38 MAPK activity. J Cell Sci. 2014 10.1242/jcs.134502 . 24481813

[29] Hughes JN, Washington JM, Zheng Z, Lau XK, Yap C, Rathjen PD, et al Manipulation of cell:cell contacts and mesoderm suppressing activity direct lineage choice from pluripotent primitive ectoderm-like cells in culture. PloS one. 2009;4(5):e5579 10.1371/journal.pone.0005579 19440553 PMC2679147

[30] Ernst M, Gearing DP, Dunn AR. Functional and biochemical association of Hck with the LIF/IL-6 receptor signal transducing subunit gp130 in embryonic stem cells. EMBO J. 1994;13(7):1574–84. 8156996 10.1002/j.1460-2075.1994.tb06420.x PMC394987

[31] Kunath T, Saba-El-Leil MK, Almousailleakh M, Wray J, Meloche S, Smith A. FGF stimulation of the Erk1/2 signalling cascade triggers transition of pluripotent embryonic stem cells from self-renewal to lineage commitment. Development. 2007;134(16):2895–902. 10.1242/dev.02880 . 17660198

[32] Stavridis MP, Lunn JS, Collins BJ, Storey KG. A discrete period of FGF-induced Erk1/2 signalling is required for vertebrate neural specification. Development. 2007;134(16):2889–94. 10.1242/dev.02858 . 17660197

[33] Burdon T, Stracey C, Chambers I, Nichols J, Smith A. Suppression of SHP-2 and ERK signalling promotes self-renewal of mouse embryonic stem cells. Dev Biol. 1999;210(1):30–43. 10.1006/dbio.1999.9265 . 10364425

[34] Bain J, Plater L, Elliott M, Shpiro N, Hastie CJ, McLauchlan H, et al The selectivity of protein kinase inhibitors: a further update. Biochem J. 2007;408(3):297–315. 10.1042/BJ20070797 17850214 PMC2267365

[35] Kong L, Deng Z, Shen H, Zhang Y. Src family kinase inhibitor PP2 efficiently inhibits cervical cancer cell proliferation through down-regulating phospho-Src-Y416 and phospho-EGFR-Y1173. Mol Cell Biochem. 2011;348(1–2):11–9. 10.1007/s11010-010-0632-1 . 21052789

[36] Hanke JH, Gardner JP, Dow RL, Changelian PS, Brissette WH, Weringer EJ, et al Discovery of a novel, potent, and Src family-selective tyrosine kinase inhibitor. Study of Lck- and FynT-dependent T cell activation. J Biol Chem. 1996;271(2):695–701. 10.1074/jbc.271.2.695 . 8557675

[37] Wu JX, Adamson ED. Kinase-negative mutant epidermal growth factor receptor (EGFR) expression during embryonal stem cell differentiation favours EGFR-independent lineages. Development. 1996;122(10):3331–42. . 8898244 10.1242/dev.122.10.3331

[38] Meyn MA 3rd, Schreiner SJ, Dumitrescu TP, Nau GJ, Smithgall TE. SRC family kinase activity is required for murine embryonic stem cell growth and differentiation. Mol Pharmacol. 2005;68(5):1320–30. 10.1124/mol.104.010231 . 15985613

[39] Meyn MA 3rd, Smithgall TE. Chemical genetics identifies c-Src as an activator of primitive ectoderm formation in murine embryonic stem cells. Sci Signal. 2009;2(92):ra64 10.1126/scisignal.2000311 19825829 PMC2775445

[40] Aouadi M, Bost F, Caron L, Laurent K, Le Marchand Brustel Y, Binetruy B. p38 mitogen-activated protein kinase activity commits embryonic stem cells to either neurogenesis or cardiomyogenesis. Stem Cells. 2006;24(5):1399–406. 10.1634/stemcells.2005-0398 . 16424397

[41] Chen S, Do JT, Zhang Q, Yao S, Yan F, Peters EC, et al Self-renewal of embryonic stem cells by a small molecule. Proc Natl Acad Sci U S A. 2006;103(46):17266–71. 10.1073/pnas.0608156103 17088537 PMC1859921

[42] Wu J, Kubota J, Hirayama J, Nagai Y, Nishina S, Yokoi T, et al p38 Mitogen-activated protein kinase controls a switch between cardiomyocyte and neuronal commitment of murine embryonic stem cells by activating myocyte enhancer factor 2C-dependent bone morphogenetic protein 2 transcription. Stem Cells Dev. 2010;19(11):1723–34. 10.1089/scd.2010.0066 . 20412016

[43] Barruet E, Hadadeh O, Peiretti F, Renault VM, Hadjal Y, Bernot D, et al p38 mitogen activated protein kinase controls two successive-steps during the early mesodermal commitment of embryonic stem cells. Stem Cells Dev. 2011;20(7):1233–46. 10.1089/scd.2010.0213 . 20954847

[44] Ying QL, Stavridis M, Griffiths D, Li M, Smith A. Conversion of embryonic stem cells into neuroectodermal precursors in adherent monoculture. Nat Biotechnol. 2003;21(2):183–6. 10.1038/nbt780 . 12524553

[45] Blake RA, Broome MA, Liu X, Wu J, Gishizky M, Sun L, et al SU6656, a selective src family kinase inhibitor, used to probe growth factor signaling. Mol Cell Biol. 2000;20(23):9018–27. 10.1128/mcb.20.23.9018-9027.2000 11074000 PMC86555

[46] Williams MR, Arthur JS, Balendran A, van der Kaay J, Poli V, Cohen P, et al The role of 3-phosphoinositide-dependent protein kinase 1 in activating AGC kinases defined in embryonic stem cells. Curr Biol. 2000;10(8):439–48. 10.1016/s0960-9822(00)00441-3 . 10801415

[47] Anneren C, Cowan CA, Melton DA. The Src family of tyrosine kinases is important for embryonic stem cell self-renewal. J Biol Chem. 2004;279(30):31590–8. 10.1074/jbc.M403547200 . 15148312

[48] Rouse J, Cohen P, Trigon S, Morange M, Alonso-Llamazares A, Zamanillo D, et al A novel kinase cascade triggered by stress and heat shock that stimulates MAPKAP kinase-2 and phosphorylation of the small heat shock proteins. Cell. 1994;78(6):1027–37. 10.1016/0092-8674(94)90277-1 . 7923353

[49] Darragh J, Soloaga A, Beardmore VA, Wingate AD, Wiggin GR, Peggie M, et al MSKs are required for the transcription of the nuclear orphan receptors Nur77, Nurr1 and Nor1 downstream of MAPK signalling. Biochem J. 2005;390(Pt 3):749–59. 10.1042/BJ20050196 15910281 PMC1199668

[50] Allen M, Svensson L, Roach M, Hambor J, McNeish J, Gabel CA. Deficiency of the stress kinase p38alpha results in embryonic lethality: characterization of the kinase dependence of stress responses of enzyme-deficient embryonic stem cells. J Exp Med. 2000;191(5):859–70. 10704466 10.1084/jem.191.5.859 PMC2195860

[51] Giannoni E, Buricchi F, Raugei G, Ramponi G, Chiarugi P. Intracellular reactive oxygen species activate Src tyrosine kinase during cell adhesion and anchorage-dependent cell growth. Mol Cell Biol. 2005;25(15):6391–403. 10.1128/MCB.25.15.6391-6403.2005 16024778 PMC1190365

[52] Cuadrado A, Nebreda AR. Mechanisms and functions of p38 MAPK signalling. Biochem J. 2010;429(3):403–17. 10.1042/BJ20100323 . 20626350

[53] Ray PD, Huang BW, Tsuji Y. Reactive oxygen species (ROS) homeostasis and redox regulation in cellular signaling. Cell Signal. 2012;24(5):981–90. 10.1016/j.cellsig.2012.01.008 22286106 PMC3454471

[54] Choi TG, Lee J, Ha J, Kim SS. Apoptosis signal-regulating kinase 1 is an intracellular inducer of p38 MAPK-mediated myogenic signalling in cardiac myoblasts. Biochim Biophys Acta. 2011;1813(8):1412–21. 10.1016/j.bbamcr.2011.04.001 . 21530592

[55] Liu W, Phang JM. Proline dehydrogenase (oxidase) in cancer. Biofactors. 2012;38(6):398–406. 10.1002/biof.1036 . 22886911 PMC7479541

[56] Hamilton WB, Kaji K, Kunath T. ERK2 suppresses self-renewal capacity of embryonic stem cells, but is not required for multi-lineage commitment. PloS one. 2013;8(4):e60907 10.1371/journal.pone.0060907 23613754 PMC3628700

[57] Shimizu T, Ueda J, Ho JC, Iwasaki K, Poellinger L, Harada I, et al Dual inhibition of Src and GSK3 maintains mouse embryonic stem cells, whose differentiation is mechanically regulated by Src signaling. Stem Cells. 2012;30(7):1394–404. 10.1002/stem.1119 . 22553165

[58] Li X, Zhu L, Yang A, Lin J, Tang F, Jin S, et al Calcineurin-NFAT signaling critically regulates early lineage specification in mouse embryonic stem cells and embryos. Cell Stem Cell. 2011;8(1):46–58. 10.1016/j.stem.2010.11.027 . 21211781

[59] Theunissen TW, Powell BE, Wang H, Mitalipova M, Faddah DA, Reddy J, et al Systematic Identification of Culture Conditions for Induction and Maintenance of Naive Human Pluripotency. Cell Stem Cell. 2014 10.1016/j.stem.2014.07.002 . 28903029 PMC5628950

[60] Vallier L, Mendjan S, Brown S, Chng Z, Teo A, Smithers LE, et al Activin/Nodal signalling maintains pluripotency by controlling Nanog expression. Development. 2009;136(8):1339–49. 10.1242/dev.033951 19279133 PMC2687465

[61] Ware CB, Wang L, Mecham BH, Shen L, Nelson AM, Bar M, et al Histone deacetylase inhibition elicits an evolutionarily conserved self-renewal program in embryonic stem cells. Cell Stem Cell. 2009;4(4):359–69. 10.1016/j.stem.2009.03.001 19341625 PMC2719860

[62] Ding VM, Boersema PJ, Foong LY, Preisinger C, Koh G, Natarajan S, et al Tyrosine phosphorylation profiling in FGF-2 stimulated human embryonic stem cells. PloS one. 2011;6(3):e17538 10.1371/journal.pone.0017538 21437283 PMC3060089

[63] Tamm C, Galito SP, Anneren C. Differential effects on cell motility, embryonic stem cell self-renewal and senescence by diverse Src kinase family inhibitors. Exp Cell Res. 2012;318(4):336–49. 10.1016/j.yexcr.2011.12.008 . 22197704

[64] Chakraborty S, Kang B, Huang F, Guo YL. Mouse embryonic stem cells lacking p38alpha and p38delta can differentiate to endothelial cells, smooth muscle cells, and epithelial cells. Differentiation. 2009;78(2–3):143–50. 10.1016/j.diff.2009.05.006 19539422 PMC2761660

[65] Toyooka Y, Shimosato D, Murakami K, Takahashi K, Niwa H. Identification and characterization of subpopulations in undifferentiated ES cell culture. Development. 2008;135(5):909–18. 10.1242/dev.017400 . 18263842

[66] Mitsui K, Tokuzawa Y, Itoh H, Segawa K, Murakami M, Takahashi K, et al The homeoprotein Nanog is required for maintenance of pluripotency in mouse epiblast and ES cells. Cell. 2003;113(5):631–42. 10.1016/s0092-8674(03)00393-3 . 12787504

[67] Hayashi K, Lopes SM, Tang F, Surani MA. Dynamic equilibrium and heterogeneity of mouse pluripotent stem cells with distinct functional and epigenetic states. Cell Stem Cell. 2008;3(4):391–401. 10.1016/j.stem.2008.07.027 18940731 PMC3847852

[68] Chambers I, Silva J, Colby D, Nichols J, Nijmeijer B, Robertson M, et al Nanog safeguards pluripotency and mediates germline development. Nature. 2007;450(7173):1230–4. 10.1038/nature06403 . 18097409

[69] Acampora D, Di Giovannantonio LG, Simeone A. Otx2 is an intrinsic determinant of the embryonic stem cell state and is required for transition to a stable epiblast stem cell condition. Development. 2013;140(1):43–55. 10.1242/dev.085290 . 23154415

[70] Furusawa T, Ohkoshi K, Honda C, Takahashi S, Tokunaga T. Embryonic stem cells expressing both platelet endothelial cell adhesion molecule-1 and stage-specific embryonic antigen-1 differentiate predominantly into epiblast cells in a chimeric embryo. Biol Reprod. 2004;70(5):1452–7. 10.1095/biolreprod.103.024190 . 14736812

[71] Yang SH, Kalkan T, Morissroe C, Marks H, Stunnenberg H, Smith A, et al Otx2 and Oct4 Drive Early Enhancer Activation during Embryonic Stem Cell Transition from Naive Pluripotency. Cell Rep. 2014;7(6):1968–81. 10.1016/j.celrep.2014.05.037 24931607 PMC4074343

[72] Hu G, Kim J, Xu Q, Leng Y, Orkin SH, Elledge SJ. A genome-wide RNAi screen identifies a new transcriptional module required for self-renewal. Genes Dev. 2009;23(7):837–48. 10.1101/gad.1769609 19339689 PMC2666338

[73] Westerman BA, Braat AK, Taub N, Potman M, Vissers JH, Blom M, et al A genome-wide RNAi screen in mouse embryonic stem cells identifies Mp1 as a key mediator of differentiation. J Exp Med. 2011;208(13):2675–89. 10.1084/jem.20102037 22143885 PMC3244037

[74] Wong DJ, Liu H, Ridky TW, Cassarino D, Segal E, Chang HY. Module map of stem cell genes guides creation of epithelial cancer stem cells. Cell Stem Cell. 2008;2(4):333–44. 10.1016/j.stem.2008.02.009 18397753 PMC2628721

[75] Kim J, Chu J, Shen X, Wang J, Orkin SH. An extended transcriptional network for pluripotency of embryonic stem cells. Cell. 2008;132(6):1049–61. 10.1016/j.cell.2008.02.039 18358816 PMC3837340

[76] Kim J, Woo AJ, Chu J, Snow JW, Fujiwara Y, Kim CG, et al A Myc network accounts for similarities between embryonic stem and cancer cell transcription programs. Cell. 2010;143(2):313–24. 10.1016/j.cell.2010.09.010 20946988 PMC3018841

[77] Nichols J, Silva J, Roode M, Smith A. Suppression of Erk signalling promotes ground state pluripotency in the mouse embryo. Development. 2009;136(19):3215–22. 10.1242/dev.038893 19710168 PMC2739140

[78] Donald SP, Sun XY, Hu CA, Yu J, Mei JM, Valle D, et al Proline oxidase, encoded by p53-induced gene-6, catalyzes the generation of proline-dependent reactive oxygen species. Cancer Res. 2001;61(5):1810–5. . 11280728

[79] Liu Y, Borchert GL, Surazynski A, Hu CA, Phang JM. Proline oxidase activates both intrinsic and extrinsic pathways for apoptosis: the role of ROS/superoxides, NFAT and MEK/ERK signaling. Oncogene. 2006;25(41):5640–7. 10.1038/sj.onc.1209564 . 16619034

[80] Lee SH, Lee YJ, Park SW, Kim HS, Han HJ. Caveolin-1 and integrin beta1 regulate embryonic stem cell proliferation via p38 MAPK and FAK in high glucose. J Cell Physiol. 2011;226(7):1850–9. 10.1002/jcp.22510 . 21506116

[81] Rathjen J, Haines BP, Hudson KM, Nesci A, Dunn S, Rathjen PD. Directed differentiation of pluripotent cells to neural lineages: homogeneous formation and differentiation of a neurectoderm population. Development. 2002;129(11):2649–61. . 12015293 10.1242/dev.129.11.2649

[82] Doetschman TC, Eistetter H, Katz M, Schmidt W, Kemler R. The in vitro development of blastocyst-derived embryonic stem cell lines: formation of visceral yolk sac, blood islands and myocardium. J Embryol Exp Morphol. 1985;87:27–45. . 3897439

[83] Rathjen J, Rathjen PD. Lineage specific differentiation of mouse ES cells: formation and differentiation of early primitive ectoderm-like (EPL) cells. Methods Enzymol. 2003;365:3–25. 10.1016/S0076-6879(03)65001-9 . 14696334

[84] Simon P. Q-Gene: processing quantitative real-time RT-PCR data. Bioinformatics. 2003;19(11):1439–40. 10.1093/bioinformatics/btg157 . 12874059

