# Supplementary figures and images for: Src Family Kinases and p38 Mitogen-Activated Protein Kinases Regulate Pluripotent Cell Differentiation in Culture
Source: PLoS One. 2016 Oct 10;11(10):e0163244. doi: 10.1371/journal.pone.0163244 (PMC5056717; doi:10.1371/journal.pone.0163244)

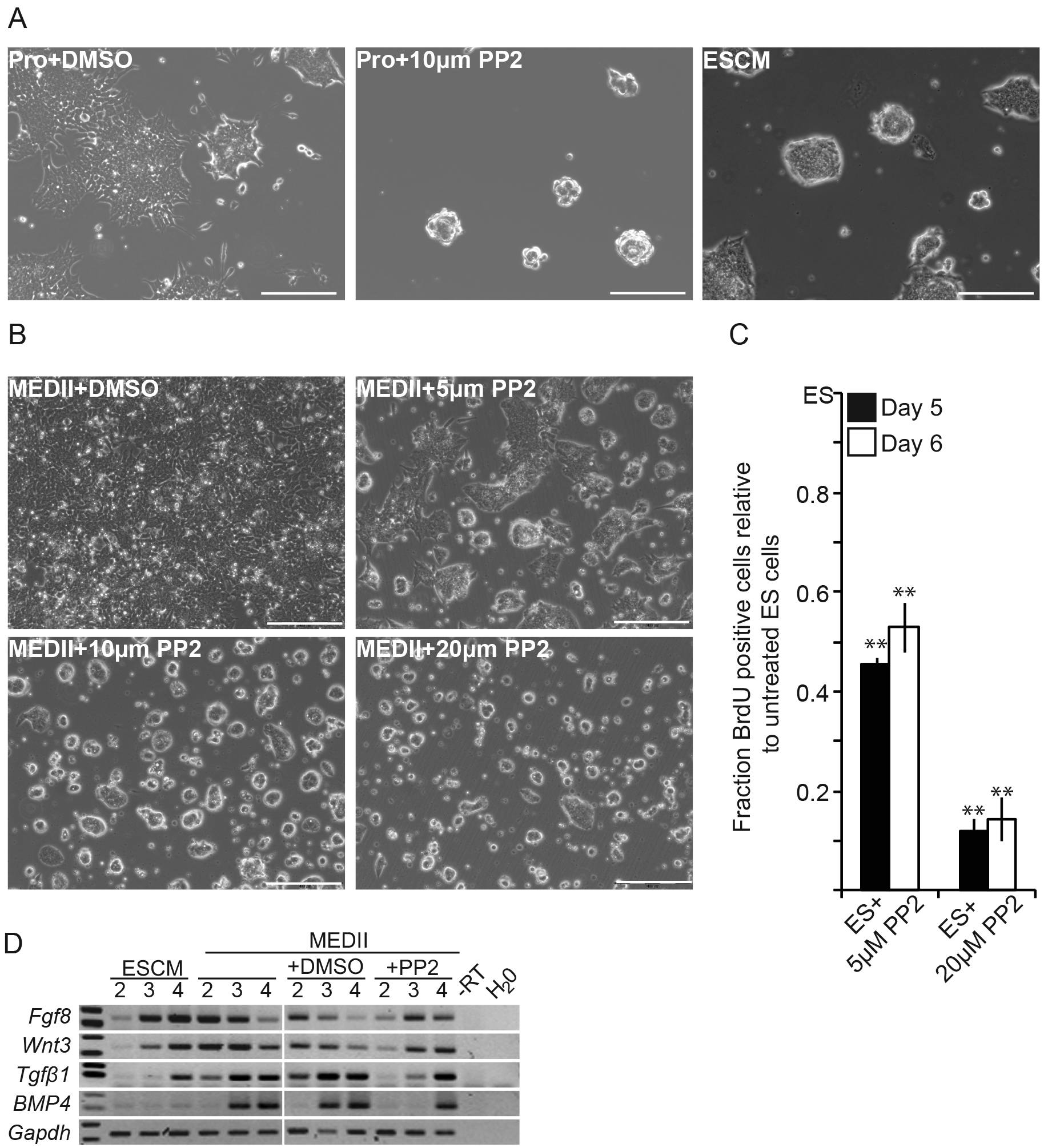

Supplement: S1 Fig — A. ES cells were cultured in ESCM and ESCM + 200 μM L-proline with or without 10 μM PP2, as indicated, for 4 days. Scale bar = 200 μm. B. ES cells were cultured in MEDII + DMSO and MEDII + 5, 10 or 20 μM PP2 for 3 days. Scale bar = 200 μm. Although a clear change in the appearance of colonies could be seen with 5 μM PP2 when compared to MEDII + DMSO, full suppression of EPL cell morphology was seen at 10 and 20 μM. C. ES cells were cultured with 5 and 20 μM PP2 for 5 (■) or 6 (□) days. Cells in S-phase were identified by immunofluorescence for incorporated BrdU followed by flow cytometry. The number of cells incorporating BrdU is shown relative to ES cells. Error bars represent SEM; n = 3. Comparisons were made to untreated ES cells, ** p ≤ 0.01. D. ES cells were cultured in ESCM, MEDII, MEDII + DMSO and MEDII + 10 μM PP2, as indicated, for 3 days and formed into EBs. EBs were collected on days 2, 3 and 4. RNA was isolated and analyzed for expression of Fgf8, Wnt3, Tgfβ1, Bmp4 and Gapdh by RT-PCR; n = 3, a representative image is shown. (TIF) [file pone.0163244.s001.tif]

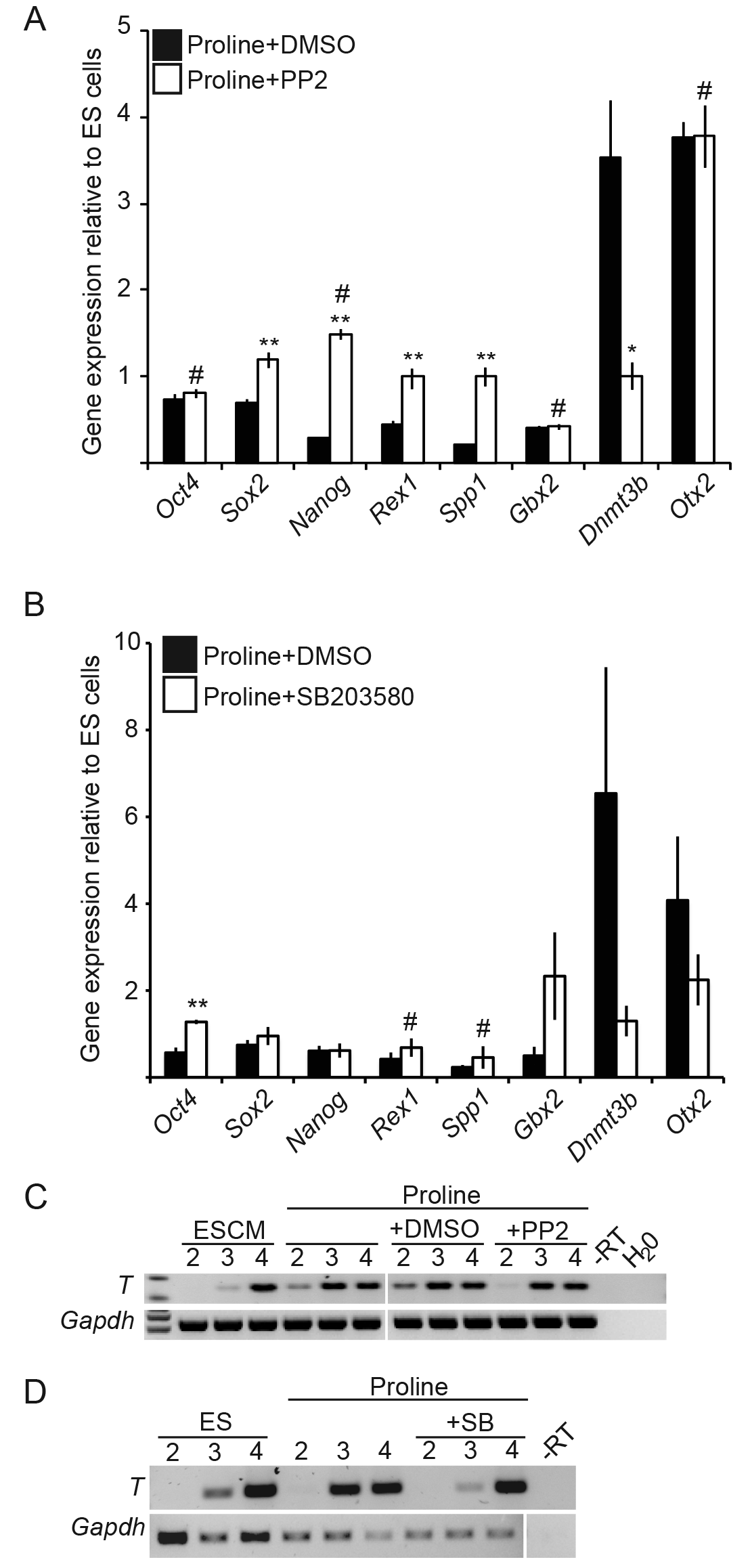

Supplement: S2 Fig — A, B. ES cells were cultured in medium supplemented with 200 μM l-proline and DMSO (■) or l-proline and 10 μM PP2 (□) (A) or 10 μM SB203580 (□)(B), for 4 days. RNA from these cells was analyzed for transcripts of Oct4, Sox2, Nanog, Rex1, Spp1, Gbx2, Dnmt3b, Otx2 and Fgf5 by real-time PCR. Expression was normalized to β-actin and expressed relative to ES cells. Error bars represent SEM; n = 3. ES cells in Proline + PP2 were compared to cells cultured in Proline + DMSO (** p ≤ 0.05) or ES cells (# p ≤ 0.05). C, D. ES cells were cultured with l-proline + DMSO, l-proline +10 μM PP2 (C) and L-proline +10 μM SB203580 (D) for 4 days before being formed into embryoid bodies (EBs). EBs were analysed as for the expression of T by RT-PCR. n = 3. (TIF) [file pone.0163244.s002.tif]

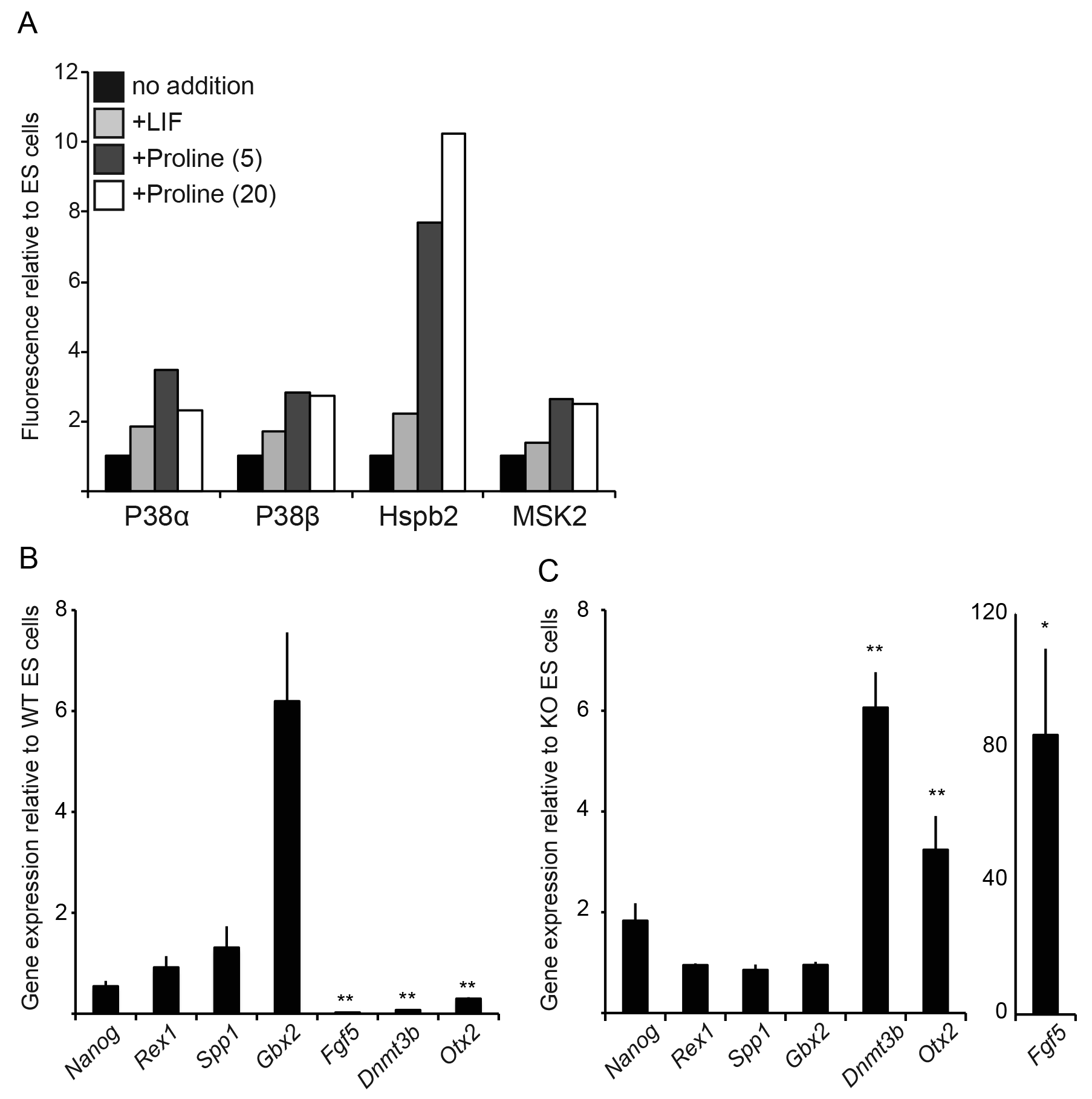

Supplement: S3 Fig — A. ES cells were treated with LIF or 100 μM of l-proline for 5 or 20 minutes, as indicated. Cells were lysed and protein bound to the kinome array and binding quantified. Binding to antibodies specific for pp38α, pp38β, pHsbp2 and MSK2 is shown; n = 2, values have been averaged. B. WT ES cell and p38α KO ES cells were cultured in ESCM. RNA was analyzed by qPCR for the expression of Nanog, Rex1, Spp1, Gbx2, Fgf5, Dnmt3b and Otx2. Expression was normalized to Oct4 and expressed relative to WT ES cells. Error bars represent SEM; n = 3. **p ≤ 0.01 when compared to WT ES cells. Loss of p38α decreased expression of primitive ectoderm markers in the ES cell population. C. p38α KO ES cells were cultured in ESCM and MEDII for 3 days to form EPL cells. RNA was analyzed by qPCR for the expression of Nanog, Rex1, Spp1, Gbx2, Fgf5, Dnmt3b and Otx2 by real-time PCR. Expression was normalized to Oct4 and expressed relative to p38α KO ES cells. Error bars represent SEM; n = 3. **p ≤ 0.01, *p ≤ 0.05 when compared to KO ES cells. (TIF) [file pone.0163244.s003.tif]

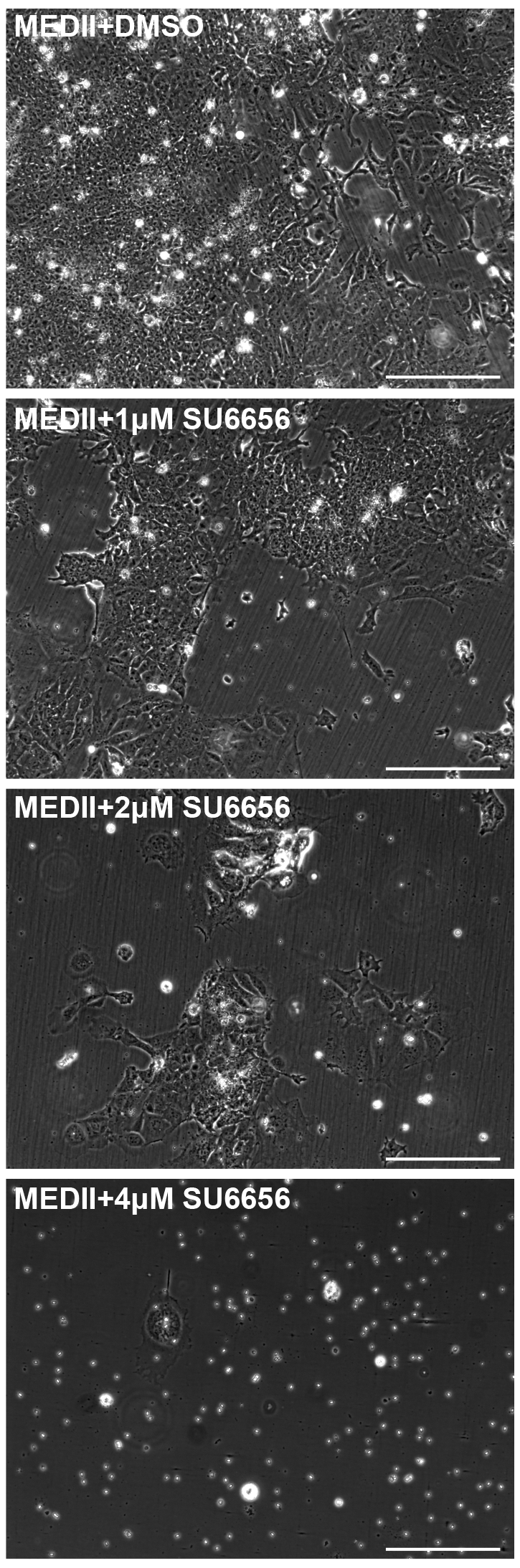

Supplement: S4 Fig — ES cells were cultured in MEDII + DMSO and MEDII + 1, 2 or 4 μM SU6656 for 3 days. Scale bar = 200 μm. (TIF) [file pone.0163244.s004.tif]
