# Supplementary material for: Src Family Kinases and p38 Mitogen-Activated Protein Kinases Regulate Pluripotent Cell Differentiation in Culture
Source: PLoS One. 2016 Oct 10;11(10):e0163244. doi: 10.1371/journal.pone.0163244 (PMC5056717; doi:10.1371/journal.pone.0163244)
Supplement: S1 Table — (DOCX) [file pone.0163244.s005.docx]

| **Table S1: Details of consumables used in this research** | | |
| --- | --- | --- |
| **Primers used for detecting gene expression** | | |
| **Gene** | **Sequence 5' > 3'** | **Amplicon (bp)** |
| *Brachyury* (*T*) | F - TGCTGCCTGTGAGTCATAAC | 143 |
|  | R - GCCTCGAAAGAACTGAGCTC |  |
| *Dnmt3b* | F - AGTGCAGACAATAACCACCAAGTC | 87 |
|  | R - ACGTCGTCCTTGCCATTCA |  |
| *Fgf5* | F - CTGCAGATCTACCCGGATG | 169 |
|  | R - TAAATTTGGCACTTGCATGG |  |
| *Gapdh* | F - CTTCACCACCATGGAGAAGGC | 236 |
|  | R - GGCATGGACTGTGGTCATGAG |  |
| *Gbx2* | F - CTCGCTGCTCGCTTTCTCT | 100 |
|  | R - CGGGTCATCTTCCAGCTTT |  |
| *Oct4* | F - CCCAGGCCGACGTGG | 65 |
|  | R - GATGGTGGTCTGGCTGAACAC |  |
| *Otx2* | F - CCGGGTTCTTTTTAGTTAGT | 488 |
|  | R - AGCTCTTCGATTCTTAAACC |  |
| *Mixl1* | F - CTTCCGACAGACCATGTA | 145 |
|  | R - GATAAGGGCTGAAATGACTTCCC |  |
| *Nanog* | F - CAGAAAAACCAGTGGTTGAAGACT | 81 |
|  | R - GCAATGGATGCTGGGATACTC |  |
| *Rex1* | F - TGCCTCCAAGTGTTGTCCC | 119 |
|  | R - ATTCATGTTGTCTTAGCTGCTTCC |  |
| *Sox1* | F - GACTTGCAGGCTATGTACAACATC | 171 |
|  | R - CCTCTCAGACGGTGGAGTTATATT |  |
| *Sox2* | F - CCCCTTTTATTTTCCGTAGTTGTAT | 151 |
|  | R - TCAAACTGTGCATAATGGAGTAAAA |  |
| *Spp1* | F - GCTTGGCTTATGGACTGAGG | 82 |
|  | R - AGGTCCTCATCTGTGGCATC |  |
| *β-actin* | F - CTGCCTGACGGCCAGG | 89 |
|  | R - GATTCCATACCCAAGAAGGAAGG |  |
| **Antibodies used for detecting protein expression** | | |
| **Antibody** | **Source / Company** | **Dilution** |
| HSP27 | Rabbit / R&D Systems; AF1580 | 1:1000 |
| p38 MAPK | Rabbit / Cell Signaling Technology; #9212 | 1:1000 |
| p44/42 MAPK (Erk1/2) | Rabbit / Cell Signaling Technology; #9102 | 1:1000 |
| Phospho-HSP27 (S78/S82) | Rabbit / R&D Systems; AF2314 | 1:1000 |
| Phospho-p38 MAPK (Thr180/Tyr182) | Rabbit / Cell Signaling Technology; #9211 | 1:1000 |
| Phospho-p44/42 MAPK (Thr202/Tyr204) | Rabbit / Cell Signaling Technology; #9101 | 1:1000 |
| Phospho-SRC (pY418) | Rabbit / Life Technologies; 44-660G | 1:1000 |
| β-Tubulin | Mouse / Sigma-Aldrich; T8328 | 1:20000 |
| Anti-mouse IgG, HRP-linked | Rabbit / DAKO; P0260 | 1:5000 |
| Anti-rabbit IgG, HRP-linked | Goat / Cell Signaling Technology; #7074 | 1:5000 |
| BrdU | Mouse / Bioclone Australia (Cat # A1-9452) | 1:200 |
| Anti-mouse IgG Alexa Fluor 488 | Rabbit / Invitrogen (Cat# A11059) | 1:1000 |
